# The Potential Vaccine Component for COVID-19: A Comprehensive Review of Global Vaccine Development Efforts

**DOI:** 10.7759/cureus.8871

**Published:** 2020-06-27

**Authors:** Israr Khan, Zahoor Ahmed, Ayesha Sarwar, Abdur Jamil, Faiz Anwer

**Affiliations:** 1 Internal Medicine, Bolan Medical College, Quetta, PAK; 2 Internal Medicine, King Edward Medical University, Mayo Hospital, Lahore, PAK; 3 Internal Medicine, Central Michigan University, Saginaw, USA; 4 Hematology and Oncology, Cleveland Clinic, Cleveland, USA

**Keywords:** coronavirus, covid-19, sars-cov-2, vaccine

## Abstract

The whole world is concerned about the pandemic of coronavirus disease (COVID-19), caused by the severe acute respiratory syndrome coronavirus-2 (SARS-CoV-2), due to fatality of this condition. This has become a public health emergency of international concern. No specific vaccine and medicine have proven effective in large-sized trials at this time. With the rapidly increasing number of positive cases and deaths, there is a dire need for effective treatments and an effective vaccine for prevention. An urgent unmet need led to the planning and opening of multiple drug development trials for treatment and vaccine development. In this article, we have summarized data on cell receptor interactions and data on prospects of new vaccines targeting the deoxyribonucleic acid (DNA), messenger ribonucleic acid (mRNA), and viral minigenes. We have tabulated the available data on various clinical trials testing various aspects of COVID-19 vaccines.

## Introduction and background

Severe acute respiratory syndrome coronavirus 2 (SARS-COV-2), the causative agent of COVID-19 (coronavirus disease-2019), first appeared in Wuhan, China by the end of December of 2019. It started as an outbreak which headed to an epidemic with 44,672 confirmed cases in China by February 14, 2020, with a 2.3% reported mortality rate. The mortality rate was comparatively lower than the previously known epidemics caused by human coronaviruses [SARS-CoV) and the Middle East Respiratory Syndrome Coronavirus (MERS-CoV)] in 2003 and 2012 respectively [1-2]. It spread outside of China to the rest of the world in no time, primarily through human-to-human transmission by respiratory droplets or possibly through the fecal-oral route, and subsequently, the World Health Organization (WHO) declared COVID-19 as a global pandemic on March 11, 2020 [3]. Currently, with the disease spread to 216 countries, there are over 6,663,304 reported confirmed cases worldwide including over 392,802 deaths as of June 5, 2020 [4]. According to the Center for Disease Control and Prevention (CDC), more than 1,891,690 confirmed cases of COVID-19 have been reported in the United States alone involving all 50 states, causing over 109,192 deaths till June 5, 2020. The infection usually presents as fever, dry cough, shortness of breath, and sore throat. The severe respiratory involvement has been observed in patients with advanced age (over age 80). The overall case fatality rate of 14.3% has been reported in this age group [5]. An increase in mortality has also been reported in patients with underlying comorbidities such as cardiovascular disease, diabetes, and chronic respiratory disease. The severity of this disease is characterized by severe pneumonia, respiratory failure requiring mechanical support, sepsis, myocardial injury, and multi-organ failure [6-7]. However, some patients may have very mild symptoms or act as asymptomatic carriers suggesting that the actual number of cases with COVID-19 may be much higher than reported [2]. Given the rapid spread of the disease, researchers have dedicated themselves to better understand the virus, disease pathophysiology, and develop effective drugs and preventive vaccines.

While new treatment options are being sought out, one of the main areas of focus has been trying to develop a vaccine to prevent and fight against COVID-19, and many vaccines are in the stage of preclinical trial; however, phase I and phase II trials have begun with vaccines using the viral DNA, micro genome, and messenger ribonucleic acid (mRNA).

## Review

Coronavirus structure and vaccine

The genome of the SARS-CoV-2 consists of single-stranded positive-sense RNA encapsulated within a membrane envelope which has glycoprotein spikes giving coronaviruses their crown-like appearance [8]. Of the four classes of coronaviruses (alpha, beta, gamma, and delta), SARS-CoV, MERS-CoV, and COVID-19 causative SARS-CoV-2 are included in the class beta. While SARS-CoV, MERS-CoV, and SARS-CoV-2, all attack the lower respiratory tract, SARS-CoV-2 additionally also affects the heart, gastrointestinal system, liver, kidney, and the central nervous system eventually leading to multi-organ failure [9-10].

Glycosylated spike (S) protein, which is one of the structural proteins encoded by the coronavirus genome, is a major inducer of host immune response. This protein binds to angiotensin-converting enzyme 2 (ACE2) receptor protein located on the host cell surface membrane and mediates the host cell invasion [11-13]. Host cell produced serine protease TMPRSS211 facilitates the priming of S protein required for this invasion process. Several other nonstructural proteins are also encoded by the viral genome. This includes RNA-dependent RNA polymerase (RdRp), papain-like protease (PLpro), and coronavirus main protease (3CLpro). Once the virus enters the host cells, the viral genome (single-stranded positive RNA) is released, which is then translated into viral polyprotein with the use of host cell protein translation machinery. Viral proteinases 3CLpro and PLpro cleave viral polyproteins into effector proteins. RdRp functions to synthesize a full-length negative-strand RNA template which is used to make more viral genomic RNA. PLpro deubiquitinates certain host cell proteins like interferon factor 3 and NF-kB, resulting in immune suppression [14-16]. There exists similarity in 95% of the sequence for RdRp and 3CLpro between SARS-CoV and SARS-CoV-2. These two viruses share 76% of sequence similarity in their S protein, a highly conserved receptor-binding domain (RBD), and a domain of S protein [17-20].

**Figure 1:**
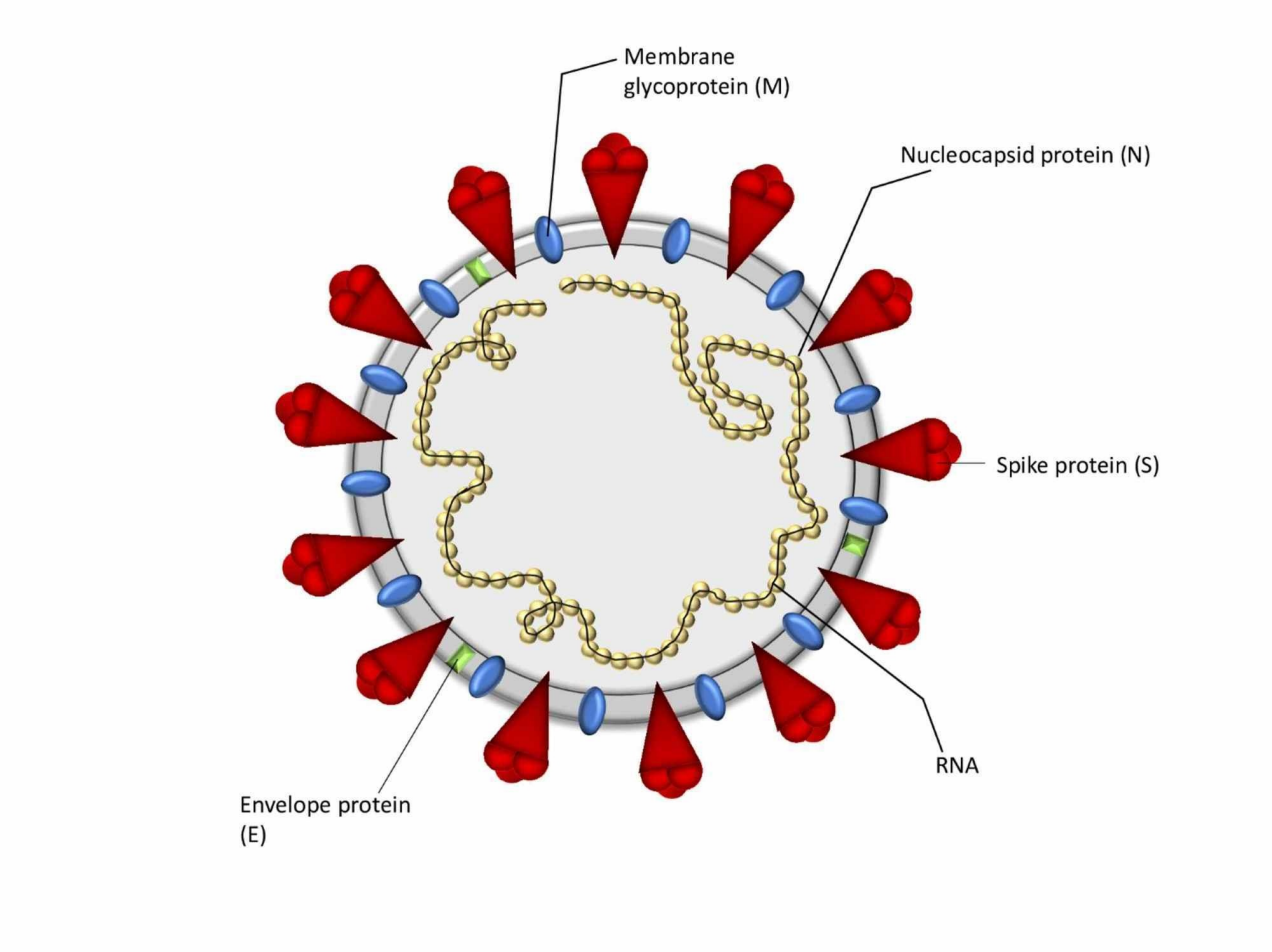
Coronavirus structure.

The novel coronavirus is an enveloped, single-stranded positive-sense RNA virus, with much of the complexity on the surface as well as within the genome (Figure 1). Envelope (E), membrane (M), nucleocapsid (N), and spike (S) are its four structural proteins while its genome comprises 29,891 nucleotides, which encode the 12 putative open reading frames responsible for the synthesis of viral structural and nonstructural proteins (Table 1) [21-22].

**Table 1:** Coronavirus structural protein and its function.

Structural protein	Function of protein
Envelope protein (E)	Interacts with M to form envelope (E)
Membrane protein (M)	Central organizer of CoV assembly and determine the shape of viral envelope
Nucleocapsid protein (N)	Binds with RNA genome to make up nucleocapsid
Spike protein (S)	Helps in entry to host cell by binding with host cell receptors

All these proteins can increase CD4+/CD8+ T-cell responses by acting as antigens to stimulate neutralizing antibodies [23]. Because of the genomic variability, researchers have currently adopted a multi-prong approach in developing effective vaccines for COVID-19, which involve: 1. whole virus vaccine, 2. recombinant subunit vaccine (genetic engineering), 3. antibody vaccine, and 4. nucleic acid vaccine [24].

Whole virus vaccines

The strategy of inactive or live attenuated whole virus vaccine has been utilized for decades due to its high immunogenicity and activation of toll-like receptors (TLRs) such as TLR3, TLR7/8, and TLR9. However, it requires long-term surveillance to evaluate its safety profile due to its live nature. A live influenza vaccine manifesting SARS-CoV-2 protein has been established by the researchers at the University of Hong Kong. Johnson & Johnson is among the few multinational companies, who are currently in the process of developing vaccines for COVID-19 [24]. Furthermore, a live attenuated coronavirus or torovirus vaccine was lately revealed by US20060039926 [25]. Similarly, efforts are underway in exploring of SARS-CoV-2 whole vaccine candidate due to the introduction of “codon deoptimization” technology for attenuating viruses by Codagneix [24].

Recombinant subunit vaccines

The process of host cell invasion mediated by interaction of spike (S) protein present on SARS-COV-1 and SARS-COV-2 and angiotensinogen converting enzyme 2 (ACE2) found on host cells membrane, is thought to be the primary trigger of host immune responses. TMPRSS211, a serine protease inhibitor produced by host cells, further augments this process via priming with (S) protein [25]. The two identified subunits of spike (S) protein responsible for generating immune responses are S1 and S2. The host cell membrane ACE2 receptor and virus interaction occur via the S1 subunit. In contrast, a fusion between virus and plasma membrane happens through the S2 subunit, which ultimately results in the release of the viral genome into the cytoplasm of host cells [24]. Due to the significant ability to neutralize antibodies, recombinant proteins and vectors containing the S1 receptor-binding domain (RBD) of spike (S) protein, can be productively used in synthesizing vaccines for COVID-19 [26].

At present, six recombinant vaccines candidates such as artiﬁcial antigen-presenting cells (aAPC) (NCT04299724), mRNA (NCT04283461), adenoviral vector-5 (NCT04313127); chimpanzee adenoviral vector ChAdOx1 (NCT04324606), DNA (NCT04336410), and a lentiviral vector (NCT04276896) are undergoing the phase-one trial to determine their safety and efficacy. Due to the safety profile, the mRNA-1273 COVID-19 vaccine has reached phase I trial in just 69 days without undergoing preclinical studies [27]. As of April 12, 2020, the adenoviral vector-five has entered into the phase II trial. Similarly, ChAdOx1 has progressed to phase I/II trial in April 2020 [27]. Rocky Mountain laboratories scientists are in collaboration with Oxford University in pursuing a chimpanzee adenovirus-vectored vaccine candidate for COVID-19 [26].

Antibody vaccines

The patients recovering from SARS-Cov-2, are manifesting neutralizing antibodies. Therefore, monoclonal antibodies (mAbs) administration can be used as a potential intervention in COVID-19 patients. A clinical trial has also reported that mAbs can effectively target various domains in MERS-CoV S-protein by binding to particular epitopes at six different sites. A satisfactory response has been achieved in mice via passive immunization of neutralizing antibodies against MERS-CoV. Therefore, it is proposed that this approach can be utilized for coronaviruses to enhance humoral responses by targeting the same spike (S) protein domains. Because SARS-CoV RBD specific-neutralizing mAbs carry a cross-neutralizing ability depending on their RBDs resemblances. Furthermore, the same cross-neutralizing SARS-CoV RBD specific-neutralizing mAbs can be explored for SARS-CoV-2 [26].

Nucleic acid vaccines

Several biotech companies are utilizing nucleic acid strategies to formulate COVID-19 vaccine candidates with heightened immune responses, especially by generating specific CD8+ T cells mediated responses against SARS-CoV-2 antigens. It consists of DNA and mRNA. These types of vaccines are synthesized by incorporating nucleic acid-containing endoplasmic reticulum (ER) polypeptide to at least one antigenic peptide or peptide/polypeptide from SARS-CoV. The DNA coated particles are then transferred via gene gun, resulting in humoral and cell-mediated immune responses in animal models. In 1993, the concept of nucleic acid vaccines yielded promising results in mice by synthesizing protective immunity against influenza; however, the results were not adequately explored in humans. While Inovio pharmaceuticals are manufacturing DNA vaccine (INO-4800) to be used in a human trial in April 2020, others are embarked on developing mRNA vaccines. A potential benefit of mRNA vaccines is due to its enhanced immunogenic properties mimicking the infectious process. To achieve this purpose, multiple mRNAs are blend into a single vaccine [24-25].

According to the records of clinicaltrials.gov, a nonrandomized, open-label, phase I trial is being conducted by the National Institute of Allergy and Infectious Diseases (NIAID) for a vaccine using mRNA, which encodes for pre-fusion spike protein of SARS-CoV-2. Shenzhen Geno-Immune Medical Institute is testing another vaccine prepared by modifying dendritic cells with lentivirus vectors expressing SARS-CoV-2 minigene and immune-modulatory genes.

Role of Bacille Calmette Guerin vaccination

Based on the comparison of a number of cases and death rates per million in about 40 countries with at least 500 cases of COVID-19, according to their Bacille Calmette Guerin (BCG) vaccination status, it has been hypothesized that mortality rates are relatively lower among countries having active BCG vaccination programs. The data suggested that countries having national BCG vaccination programs had a significantly lower number of cases as well as death rates (p < 0.0001 and p < 0.0058 respectively) as compared to those which did not have or had ceased these programs. Multiple clinical trials are underway to test the effectiveness of the BCG vaccine for the prevention of COVID-19 [28].

Interim results of ongoing vaccine phase I clinical trial

Due to the rapidly escalating and evolving COVID-19 situation, efforts are underway to develop effective vaccines against SARS-CoV-2. Recently two of the ongoing vaccine trials have reached phase I and yielded significant results. An interim result from open-label randomized double-blinded clinical trial recently published on May 22, 2020, in the Lancet journal, reported by Zhu et al. 2020, holds great promise. Both humoral and T cells immune responses were noticed in just 14 days after using a single dose of new adenovirus type-5 vectored COVID-19 (Ad5-nCoV) in healthy individuals (n=108) in the age ranges from 18 to 60. Participants were divided into three groups, such as low dose group (n=36), medium dose group (n=36), and high dose group (n=36). Some binding antibodies (Abs) (Abs that bind but do not necessarily neutralize coronavirus) titers were observed within 14 days of vaccine administration. The response rate was 16/36 (44%) in the low dose group, 18/36 (50%) in the medium dose, and 22/36 (61%) in the high dose group. However, some volunteers demonstrated some level of neutralizing antibodies during the same period. Furthermore, T-cell mediated immune response was also shown in most of the participants, particularly in medium and high dose groups (e.g., 83.3% in low dose group, 97.2% in both medium and high dose groups) [29]. The results were way more pressing after 28 days of follow up. Binding Abs levels were 35/36 (97%), 34/36 (94%), and 36/36 (100%) in low dose group, medium dose group, and high dose group, respectively. Furthermore, neutralizing Abs against SARS-CoV-2 was also detected in 18/36 (50%) in each low and medium dose, and 27/36 (75%) in high dose group also revealed neutralizing Abs against SARS-CoV-2 [29]. 

In summary, the detectable overall immune response (both humoral and T-cell mediated immune responses against SARS-CoV-2) after 28 days of vaccination are: low dose group=28/36 (78%), medium-dose group 33/36 (92%), and high dose group=36/36 (100%). As for a safety profile is concerned, the vaccine was well tolerated at all doses within 28 days period. There were no reported serious adverse events (AEs). Although severe symptoms such as fever, fatigue, shortness of breath, and muscle pain that lasted <48 hours, had been reported in one participant in the higher dose group. However, most of the AEs were mild to moderate in nature and the most commonly encountered were mild pain at the injection site (58/108, 54%), fatigue (47/108, 44%), fever (50/108, 46%), headache (42/108, 39%), and muscle pain (18/108, 17%). 

To further evaluate vaccine safety and efficacy at six months, a phase-two trial of the Ad5-nCoV vaccine has already been launched in Wuhan, China, enrolling n=500 healthy adults, which are divided into -- middle dose (n=250), low dose ( n=125), and placebo as a control (n=125). Adults older than 60 will be participating for the first time in the phase II [29].

Interim results from another phase I trial initiated by Moderna, in March 2020, have also been reported. In this ongoing trial, participants (n=105) aged 18-55 are included. Two shots at 25 micrograms (mcg) or 100 mcg (approximately 28 days apart) will be given to each participant. Moderna shared the antibodies (Abs) data of individuals (n=8) (i.e., 25 mcg arm n=4, 100 mcg arm n=4). The detectable titer of neutralized Abs was the same as seen in recovered COVID-19 patients; however, the level was higher in 100 mcg arm than recovered patients. As per company statement, AEs have been noticed, but they “have been transient and self-resolving.” Moderna has got FDA approval for phase II. Moreover, the phase III trial is expected to be initiated in July [30]. 

AstraZeneca, another phase I/II trial, started in May 2020. Currently, enrolling 1000 adults in the age ranges from 18 to 55 years. It evaluates the safety and efficacy of AZD1222, formerly known as ChAdOx1 nCoV-19 [31]. There are also many ongoing clinical trials underway suggesting that the scientific community is working hard to overcome this problem. The candidate vaccine and its component under development have been summarized in Table 2. 

 

**Table 2:** Vaccines under development using various components of SARS-CoV-2. *Source*: WHO:  https://www.who.int/blueprint/priority-diseases/key-action/novel-coronavirus-landscape-ncov.pdf?ua=1 )

Viral product used in pre-clinical vaccine trials	Developer
DNA	Inovio Pharmaceuticals Takis/Applied DNA Sciences/Evvivax Zydus Cadila
Inactivated viral products	Sinovac Codagenix/Serum Institute of India
Nonreplicating viral vector	GeoVax/BravoVax Greffex University of Oxford Altimmune Vaxart Janssen Pharmaceutical Companies
Viral protein sub-unit	ExpreS2ion WRAIR/USAMRIID Clover Biopharmaceuticals Inc./GSK Vaxil Bio AJ Vaccines Generex/EpiVax EpiVax/Univ. of Georgia Sanofi Pasteur Novavax Heat Biologics/University of Miami University of Queensland/GSK Baylor College of Medicine iBio/CC-Pharming VIDO-InterVac University of Saskatchewan
RNA	Fudan University/Shanghai JiaoTong University/RNACure Biopharma China CDC/Tongji University/Stermina Moderna/NIAID Arcturus/Duke-NUS Imperial College London CureVac BioNTech/Fosun Pharma/Pfizer BIOCAD Sanofi Pasteur/ Translate Bio

## Conclusions

Collaborative global efforts are underway to control the COVID-19 pandemic. Currently, vaccines using viral DNA, mRNA, and micro genes are already being tested in phase I and phase II clinical trials. Researchers are identifying effective and suitable vaccine candidates and therapeutics for controlling severe COVID-19. There are no effective vaccines or specific antiviral drugs against COVID-19. Hence, we have to rely exclusively on enforcing strict preventive and control measures that reduce the risk of possible disease transmission. The scientists are trying to overcome the vaccine development challenges like vaccine safety, providing long term protection, and protecting other people. Results obtained from the recently conducted clinical trials on vaccine development are promising. Although research is in progress to improve prevention, treatment, and control of COVID-19, the documented clinical data on different approaches to treatment and prevention for COVID-19 are scarce. Further research should be directed toward the study of SARS-CoV-2 in suitable animal models for analyzing replication, transmission, and pathogenesis. Efficacy and safety-related data are urgently needed from highly coordinated randomized clinical trials.
